# Madecassoside protects retinal pigment epithelial cells against hydrogen peroxide-induced oxidative stress and apoptosis through the activation of Nrf2/HO-1 pathway

**DOI:** 10.1042/BSR20194347

**Published:** 2020-10-14

**Authors:** Jinzi Zhou, Fenghua Chen, Aimin Yan, Xiaobo Xia

**Affiliations:** 1 Department of Ophthalmology, The First People’s Hospital of Guiyang, Guiyang, Guizhou 550002, China; 2 Department of Ophthalmology, Xiangya Hospital Central South University, Changsha, Hunan 410008, China

**Keywords:** Age‐related macular degeneration (AMD), apoptosis, madecassoside (MADE), Nrf2/HO-1 signaling pathway, oxidative stress, retinal pigment epithelium (RPE)

## Abstract

Age-related macular degeneration (AMD) is a progressive and degenerative ocular disease associated with oxidative stress. Madecassoside (MADE) is a major bioactive triterpenoid saponin that possesses antioxidative activity. However, the role of MADE in AMD has never been investigated. In the current study, we aimed to evaluate the protective effect of MADE on retinal pigment epithelium (RPE) cells under oxidative stress condition. We used hydrogen peroxide (H_2_O_2_) to induce oxidative damage in human RPE cells (ARPE-19 cells). Our results showed that H_2_O_2_-caused significant decrease in cell viability and increase in lactate dehydrogenase (LDH) release were dose-dependently attenuated by MADE. MADE treatment also attenuated H_2_O_2_-induced reactive oxygen species (ROS) and malondialdehyde (MDA) production in RPE cells. The reduced glutathione (GSH) level and superoxide dismutase (SOD) activity in H_2_O_2_-induced ARPE-19 cells were elevated after MADE treatment. MADE also suppressed caspase-3 activity and bax expression, as well as increased bcl-2 expression. Furthermore, H_2_O_2_-induced increase in expression levels of HO-1 and nuclear Nrf2 were enhanced by MADE treatment. Finally, knockdown of Nrf2 reversed the protective effects of MADE on H_2_O_2_-induced ARPE-19 cells. In conclusion, these findings demonstrated that MADE protected ARPE-19 cells from H_2_O_2_-induced oxidative stress and apoptosis by inducing the activation of Nrf2/HO-1 signaling pathway.

## Introduction

Age-related macular degeneration (AMD) is a progressive and degenerative ocular disease that affects the macular region of the retina [1]. AMD is a leading cause of severe and permanent visual impairment and blindness in the world with an aging population [2]. By 2020, the number of people diagnosed with AMD is expected to be 200 million globally, and it is proposed to reach nearly 300 million by 2040 [3]. Therefore, the disease presents a serious social and economic problem. Although the pathogenesis of AMD has not been completely understood, dysfunction of retinal pigment epithelium (RPE) plays a central role in the AMD progression and is an important feature of AMD [4,5].

The eye is an exceptional organ due to its continuous exposure to environmental stimuli such as radiation, chemicals, and atmospheric oxygen [6]. Under normal conditions, these stimuli cause oxidative stress, which can be eliminated by antioxidant system. However, in the aging populations, age-mediated oxidative stress and age-dependent decline in the level of antioxidants lead to protein modifications and oxidation, contributing to the RPE dysfunction [7,8]. Over the last decade, growing body of studies prove that oxidative stress plays a crucial role in AMD development and progression [9]. Therefore, attenuating oxidative stress might be effective for prevention or treatment of AMD.

Madecassoside (MADE) is a major bioactive triterpenoid saponin isolated from *Centella asiatica* that has been found to exert various pharmacological activities including antioxidative effect [10–12]. MADE was reported to have the reactive oxygen species (ROS) scavenging activity [11,13]. MADE exerts protective effect on hydrogen peroxide (H_2_O_2_)-induced oxidative stress and autophagy in human melanocytes [10]. MADE protects human umbilical vein endothelial cells (HUVECs) from H_2_O_2_-induced oxidative injury [14]. In addition, MADE protects against d-galactose-induced cognitive impairment, which is mainly due to its ability to reduce oxidative damage [15]. However, the role of MADE in AMD has never been investigated.

In the current study, we evaluated the protective effect of MADE on human-derived RPE cell line (ARPE-19 cells) under oxidative stress conditions. The results showed that MADE protected ARPE-19 cells from H_2_O_2_-induced oxidative injury through Nrf2/HO-1 signaling pathway.

## Materials and methods

### Cell culture and treatments

Human RPE cell line ARPE-19 (American Type Culture Collection, Manassas, CA, U.S.A.) was cultured in DMEM/F12 medium (Invitrogen, Carlsbad, CA). The medium was supplemented with 10% fetal bovine serum (FBS; Invitrogen), 100 U/ml penicillin (Sigma–Aldrich, St. Louis, MO, U.S.A.), and 100 μg/ml streptomycin (Sigma–Aldrich). The cells were cultured at 37°C in humidified condition with 5% CO_2_. The cells were used for experiments at passage 3. For the H_2_O_2_ treatment groups, cells were exposed to 300 μM H_2_O_2_ for 24 h. For the MADE treatment groups, cells were treated with various concentrations of MADE (≥98% purity; Sigma–Aldrich).

### Small interfering RNA transfection

Duplex small interfering RNAs (siRNAs) for Nrf2 (si1-Nrf2: 5′-CCCTGGTCCTGTGAGAGGTAGATAT-3′ and si2-Nrf2: 5′-CCGACGGGAGTTCATTGACCTGTTA-3′) and negative control siRNA (si-NC: 5′ CCCTGGTCCCCCGAGAAAAACCCC-3′) were designed and synthesized by GenePharma (Shanghai, China). The ARPE-19 cells were seeded in a 12-well plate at the density of 1 × 10^5^ cells/well and incubated in serum-free medium for 12 h before transfection. Then, the Lipofectamine 2000 Transfection Reagent (Invitrogen) was used for the transfection according to the manufacturer’s instructions. After 48 h post-transfection, Nrf2 protein levels were validated by Western blotting.

### Cell viability assay

The effect of MADE on the ARPE-19 cells viability was determined using the cell counting kit-8 (CCK-8; Dojindo, Kumamoto, Japan) according to the manufacturer’s instructions. ARPE-19 cells were plated in 96-well plates at a density of 1 × 10^5^ cells per well. After 24-h incubation, the medium was added with different concentrations (0, 6.25, 12.5, or 25 μM) of MADE for 2 h, then stimulated with H_2_O_2_ for 24 h. Afterward, 10 μl CCK-8 was added to each well and incubated for 4 h. The optical density at 450 nm was read using a multifunctional microplate reader (Molecular Devices, Sunnyvale, CA). All the experiments were performed in triplicate.

### Lactate dehydrogenase (LDH) assay

ARPE-19 cells (1 × 10^5^ cells/well) were pretreated with different concentrations of MADE (0, 6.25, 12.5, or 25 μM) for 2 h, followed by stimulation with H_2_O_2_ for 24 h in the presence of MADE. Then the cell culture supernatant was collected for the detection of LDH content by an LDH Cytotoxicity Assay Kit (Jiancheng Biotech, Nanjing, China). All the experiments were performed in triplicate.

### Measurement of intracellular ROS generation

The production of ROS was determined through detecting the fluorescent intensity of dichlorofluorescein (DCF), which was generated by 2′,7′-dichlorofluorescein diacetate (DCFH-DA) in the presence of ROS. Briefly, ARPE-19 cells (1 × 10^4^ cells/well) were pretreated with different concentrations of MADE (0, 6.25, 12.5, or 25 μM) for 2 h, followed by stimulation with H_2_O_2_ for 24 h in the presence of MADE. Then, ARPE-19 cells were washed three times with PBS, and then incubated with 10 mM DCFH-DA in the dark for 30 min at 37°C. The DCF fluorescence was detected using SpectraMax M2 Microplate Reader (Molecular Devices, Sunnyvale, CA, U.S.A.) at excitation and emission wavelengths of 488 and 525 nm, respectively. All the experiments were performed in triplicate.

### Detection of superoxide dismutase activity, malondialdehyde, and glutathione levels

ARPE-19 cells were cultured in six-well plates (1 × 10^4^ cells/well) for 24-h incubation, following which the cells were subjected to different concentrations of MADE for 2 h and then exposed to H_2_O_2_ for 24 h. The superoxide dismutase (SOD) activity and the levels of malondialdehyde (MDA) and glutathione (GSH) were determined by using the commercially available diagnostic kits (Jiancheng Bioengineering Institute, Nanjing, China). All the experiments were performed in triplicate.

### Caspase-3 activity assay

ARPE-19 cells were cultured in six-well plates (3 × 10^3^ cells/well) for 24-h incubation, following which the cells were subjected to different concentrations of MADE for 2 h and then exposed to H_2_O_2_ for 24 h. The supernatant of the treated cells was collected and measured using a Caspase Apoptosis Assay Kit (Geno Technology, St. Louis, MO, U.S.A.) following the manufacturer’s instructions. All the experiments were performed in triplicate.

### Western blot analysis

Cytoplasmic and nuclear extracts were prepared using an NE-PER Nuclear and Cytoplasmic Extraction Reagents kit (Pierce Biotechnology, Rockford, IL, U.S.A.), following the manufacturer’s instructions. Cells were collected, washed and lysed with RIPA lysis buffer (Beyotime). The cellular lysate was centrifuged at 20000×***g*** for 20 min in 4°C, and supernatant was collected. Protein concentration in the samples was detected by bicinchoninic acid assay kit (Beyotime). Protein samples were loaded on 12% SDS/PAGE and then transferred to polyvinylidene difluoride membranes (Millipore, Bedford, MA, U.S.A.). After blocking by 5% bovine serum albumin (BSA) solution, the membranes were incubated with primary antibodies against bcl-2, bax, rabbit anti-Nrf2 (ab137550), lamin B1, HO-1, or β-actin (Abcam, Cambridge, MA, U.S.A.) overnight at 4°C. Membranes were washed and subsequently incubated with horseradish peroxidase-conjugated secondary antibody (1:3000; Abcam) at 37°C for 1 h. Bands were visualized by ECL kit (Advansta, Menlo Park, CA, U.S.A.) and analyzed using ImageJ software (Bethesda, MD, U.S.A.). The absorbance values of the target proteins were performed through Gel-Pro Analyzer version 4.0 software (Media Cybernetics, Silver Spring, MD, U.S.A.). All the experiments were performed in triplicate.

### Statistical analysis

Results were generated from three independent experiments and expressed as mean ± SEM. Experimental data were analyzed using SPSS 11.0 software (SPSS, Inc., Chicago, IL, U.S.A.) by one-way ANOVA followed by Bonferroni correction. *P*<0.05 was considered to be significantly different.

## Results

### MADE improved cell viability in H_2_O_2_-induced ARPE-19 cells

**Figure 1:**
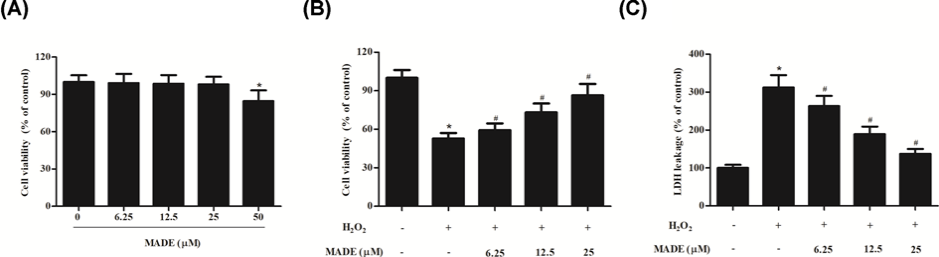
Effect of MADE on cell viability and LDH release in H_2_O_2_-induced ARPE-19 cells (**A**) ARPE-19 cells were treated with 6.25, 12.5, 25, or 50 μM MADE for 24 h. Cell viability was assessed using CCK-8 assay. (**B**) ARPE-19 cells were pre-treated with 6.25, 12.5, or 25 μM MADE for 2 h and then exposed to 300 μM H_2_O_2_ for 24 h. Cell viability was assessed using CCK-8 assay. (**C**) LDH release was measured to assess cell injury. *n*=5. *P<0.05 vs. control group; ^#^*P*<0.05 vs. H_2_O_2_ group.

First, we examined the effect of MADE on cell cytotoxicity, and the results showed that MADE was not cytotoxic to ARPE-19 cells at concentrations of less than 25 μM (Figure 1A). Then, to assess the protective influence of MADE on H_2_O_2_-induced cell injury in ARPE-19 cells, the cells were pre-treated with MADE for 2 h and then exposed to H_2_O_2_ for 24 h. H_2_O_2_ treatment resulted in a marked decrease in the cell viability in comparison with control cells. Pre-treated with MADE caused a dose-dependently increase in cell viability in comparison with H_2_O_2_-induced ARPE-19 cells (Figure 1B). In addition, the increased level of LDH in H_2_O_2_-induced ARPE-19 cells was suppressed by MADE in a dose-dependent manner (Figure 1C).

### MADE inhibited H_2_O_2_-induced oxidative stress in ARPE-19 cells

**Figure 2:**
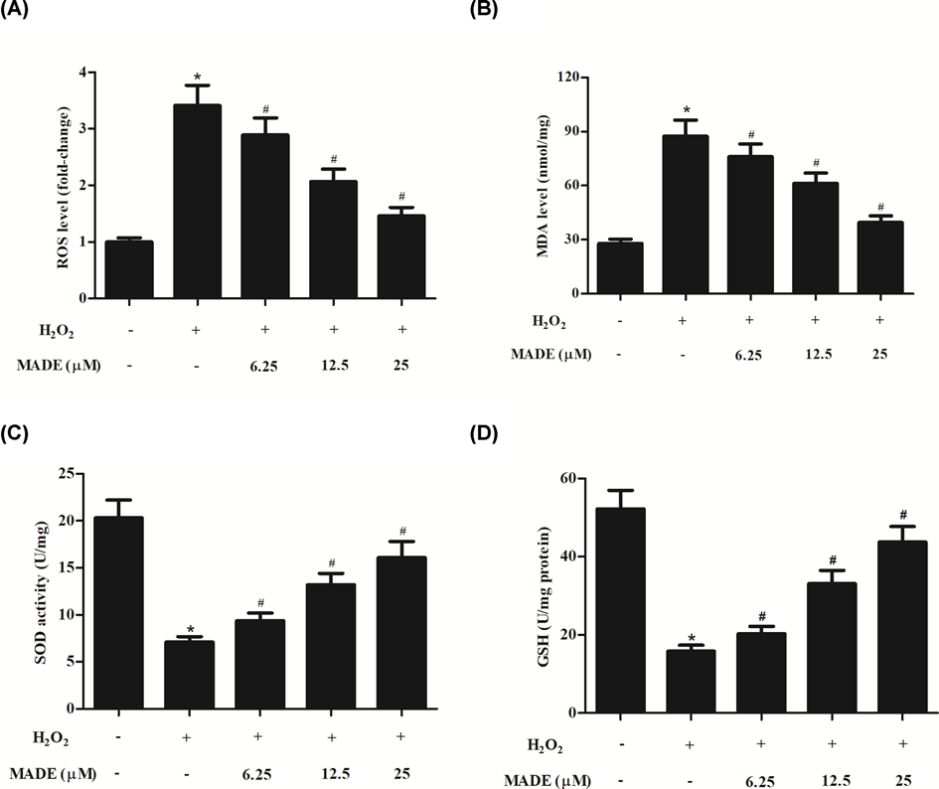
Effect of MADE on H_2_O_2_-induced oxidative stress in ARPE-19 cells The production of ROS (**A**) and MDA (**B**), as well as the SOD activity (**C**) and GSH level (**D**) were measured to reflect the degree of oxidative stress. *n*=5. *P<0.05 vs. control ARPE-19 cells. ^#^*P*<0.05 vs. ARPE-19 cells induced by H_2_O_2_ for 24 h.

To evaluate the degree of oxidative stress, the production levels of ROS, MDA, and GSH, as well as the SOD activity were determined. The production of ROS and MDA were markedly increased in ARPE-19 cells in response to H_2_O_2_. Pretreatment with MADE significantly attenuated the increased levels of ROS and MDA in H_2_O_2_-induced ARPE-19 cells (Figure 2A,B). Besides, the SOD activity and GSH level were dramatically decreased in H_2_O_2_-induced ARPE-19 cells, which were reversed by pretreatment with MADE (Figure 2C,D).

### MADE inhibited H_2_O_2_-induced apoptosis in ARPE-19 cells

**Figure 3:**
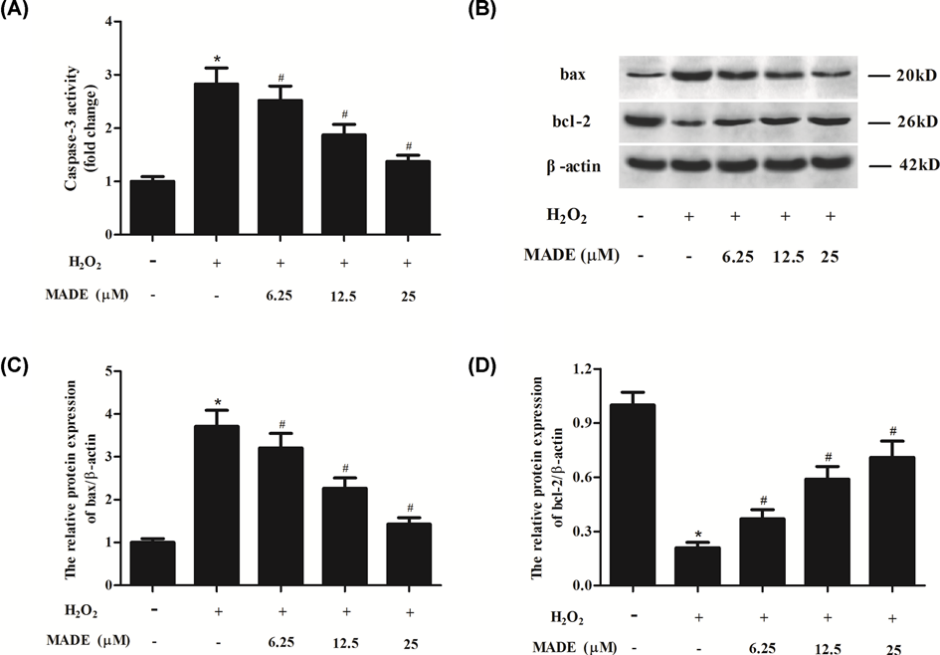
Effect of MADE on H_2_O_2_-induced apoptosis in ARPE-19 cells (**A**) The caspase-3 activity was determined. (**B**) Western blot analysis was performed to detect the expression level of pro-apoptotic Bcl-2 protein bax and anti-apoptotic protein bcl-2. β-actin was used as control protein. (**C,D**) Quantification analysis of bax and bcl-2. *n*=4. *P<0.05 vs. control ARPE-19 cells. ^#^*P*<0.05 vs. ARPE-19 cells induced by H_2_O_2_ for 24 h.

To investigate the effect of MADE on H_2_O_2_-induced apoptosis, the caspase-3 activity was determined. As shown in Figure 3A, caspase-3 activity was markedly increased by H_2_O_2_ exposure. MADE pretreatment significantly decreased the caspase-3 activity in a dose-dependent manner (Figure 3A). Next, we used Western blot to detect the expression levels of bax and bcl-2. The bax expression was up-regulated, while bcl-2 expression was down-regulated in H_2_O_2_-induced ARPE-19 cells. However, while, MADE pretreatment effectively increased Bcl-2 expression and reduced Bax expression in ARPE-19 cells (Figure 3B–D).

### MADE induced the activation of Nrf2/HO-1 pathway in ARPE-19 cells exposed to H_2_O_2_

**Figure 4:**
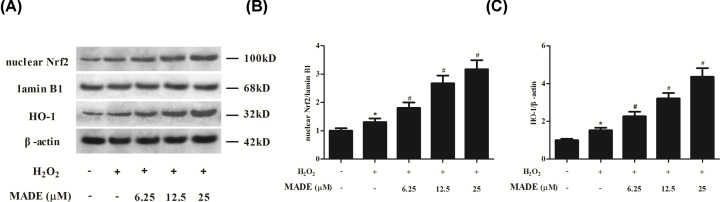
Effect of MADE on the activation of Nrf2/HO-1 pathway in H_2_O_2_-induced ARPE-19 cells (**A**) The expression levels of HO-1 and nuclear Nrf2 were measured using Western blot. β-actin and lamin B1 were respectively used as control proteins. (**B**) The ratio of nuclear Nrf2/lamin B1. (**C**) The ratio of HO-1/β-actin. *n*=4. *P<0.05 vs. control ARPE-19 cells. ^#^*P*<0.05 vs. ARPE-19 cells induced by H_2_O_2_ for 24 h.

Nrf2/HO-1 pathway is a well-known signaling involved in oxidative stress. We found that expression levels of HO-1 and nuclear Nrf2 were increased in H_2_O_2_-induced ARPE-19 cells in comparison with control cells. However, the increased expression levels of HO-1 and nuclear Nrf2 were enhanced by MADE treatment (Figure 4).

### Knockdown of Nrf2 reversed the protective effects of MADE on ARPE-19 cells

**Figure 5:**
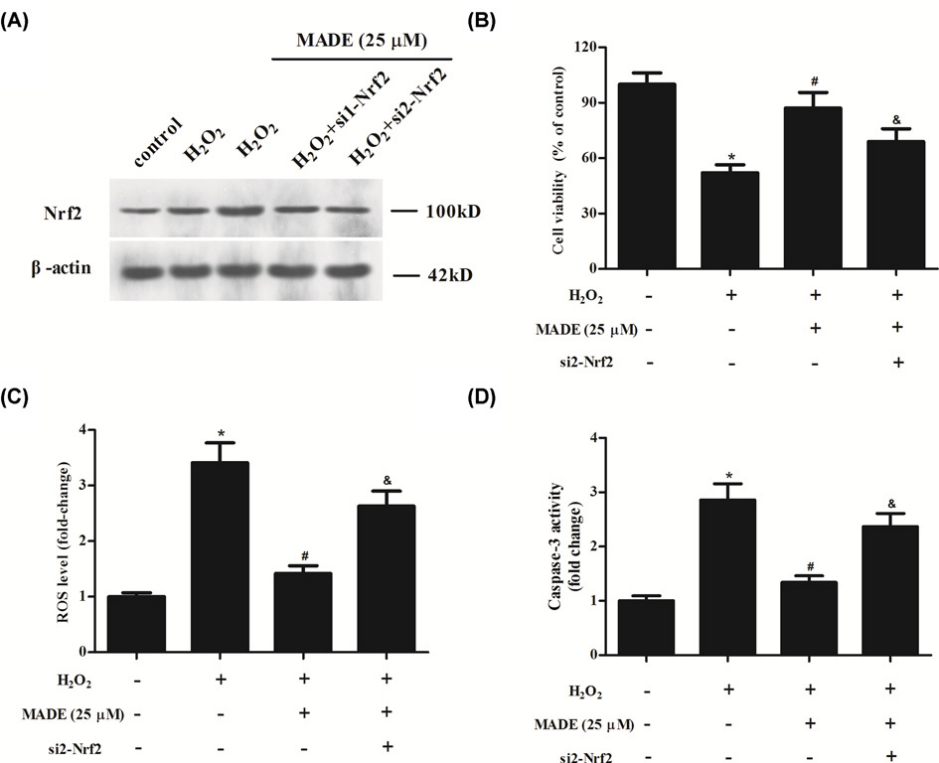
Effect of Nrf2 silencing on the protective effects of MADE on ARPE-19 cells (**A**) The knockdown of Nrf2 was examined using Western blot analysis after transfection with si1/2-Nrf2. (**B**) Cell viability was assessed using CCK-8 assay. (**C**) Level of ROS in ARPE-19 cells. (**D**) Caspase-3 activity in ARPE-19 cells. *n*=3. *P<0.05 vs. control group; ^#^*P*<0.05 vs. H_2_O_2_ group; ^&^*P*<0.05 vs. H_2_O_2_+MADE group.

To further confirm the role of Nrf2/HO-1 signaling pathway, ARPE-19 cells were transfected with si1/2-Nrf2 to silence Nrf2. The knockdown of Nrf2 was examined using Western blot analysis. Because of the higher transfection efficiency of si2-Nrf2, we selected si2-Nrf2 in the following experiments (Figure 5A). Furthermore, we found that silencing of Nrf2 partially reversed the protective effects of MADE on ARPE-19 cells with decreased cell viability, increased ROS level and caspase-3 activity (Figure 5B–D).

## Discussion

The RPE is a highly specialized, unique polarized epithelial cell that interacts with photoreceptors. Due to its remarkable and diverse functions, RPE is pivotal for maintaining normal vision [16]. With aging conditions, the RPE can become dysfunctional and die, which plays a central role in AMD pathobiology [5]. Oxidative stress has long been considered as a major phenomenon associated with aging [17]. Oxidative stress refers to a condition in which ROS levels accumulate over the extent of antioxidant defenses. The aging process is associated with the increase in ROS generation, as well a diminished antioxidant capacity and an impaired adaptive induction of antioxidants, causing oxidative modifications of macromolecules and apoptosis ensues [18,19]. As a consequence, aging-mediated oxidative stress in RPE cells plays a major role in AMD pathogenesis and progression [20]. Therefore, in the current study, we used RPE cells to evaluate the protective effect of MADE.

Among the various ROS, H_2_O_2_ has been identified as a suitable second messenger molecule that can mediate various cellular effects. Notably, overproduction of H_2_O_2_ is observed as a central hub in redox signaling and oxidative stress [21]. Hence, H_2_O_2_ is commonly used to induce oxidative stress for *in vitro* experiments. In the present study, we used H_2_O_2_ to induce oxidative damage in RPE cells. We found that cell viability was dramatically decreased, while the LDH release was significantly increased after H_2_O_2_ induction. The effects of H_2_O_2_ on cell viability and LDH release were mitigated by MADE. Besides, MADE treatment also attenuated H_2_O_2_-induced ROS production in RPE cells. MDA is the end product of the lipid peroxidation and serves as a reliable marker of oxidative stress [7]. The increased level of MDA in H_2_O_2_-induced RPE cells was reduced by MADE treatment. Endogenous antioxidants, including non-enzymatic scavenger GSH, and antioxidant enzymes such as SOD, glutathione peroxidase (GPx), and catalase (CAT), are the first lines of defense against oxidative stress and act by scavenging excessive ROS [22]. Our results showed that the GSH level and SOD activity were decreased by H_2_O_2_ induction in RPE cells. However, MADE treatment elevated the GSH level and SOD activity in H_2_O_2_-induced RPE cells. These findings suggested that MADE treatment reversed H_2_O_2_-induced oxidative stress in RPE cells.

It is well-known that ROS-mediated oxidative stress may develop RPE cells apoptosis. Excess cellular levels of ROS can lead to activation of cell death processes such as apoptosis [23]. It is documented that the main mechanism of ROS-mediated cell apoptosis is activation of the mitochondrial (intrinsic) apoptotic pathway [24]. ROS is implicated in the activation of tumor suppressor p53 and/or c-Jun N-terminal kinase (JNK), which activates pro-apoptotic Bcl-2 proteins that can inhibit the functions of anti-apoptotic proteins. After a series of reaction, caspase-9 is activated and then results in the activation of effector caspases such as caspase-3, leading to cleavage of cellular proteins and cell demise by apoptosis [23,25]. Our results showed that MADE treatment suppressed the expression of pro-apoptotic Bcl-2 protein bax, and induced the expression of anti-apoptotic protein bcl-2 in H_2_O_2_-induced RPE cells. Besides, the activation of caspase-3 in H_2_O_2_-induced RPE cells was prevented by MADE treatment. The results indicated that MADE prevented H_2_O_2_-induced cell apoptosis through inhibition of mitochondrial apoptotic pathway.

The majority of the enzymatic antioxidant defenses are regulated at transcriptional levels by the transcription factor Nrf2 [26]. Previous studies have proven that the maintenance of RPE redox homeostasis relies on the activation of the Nrf2. In the aging RPE, high amount of ROS is produced in the retina, while a decline in the antioxidant capacity is observed via a reduction in Nrf2 signaling [27]. Mounting evidence suggests that Nrf2 activation can protect the RPE from oxidative damage, which indicates the therapeutic potential of Nrf2 in the treatment of AMD [28]. Our study proved that MADE enhanced the activation of Nrf2/HO-1 pathway in ARPE-19 cells exposed to H_2_O_2_. Furthermore, knockdown of Nrf2 reversed the protective effects of MADE on H_2_O_2_-induced ARPE-19 cells, indicating that the protective role of MADE was mediated by Nrf2/HO-1 signaling pathway.

There existed several limitations in the present study. First, we only used H_2_O_2_ to induce oxidative stress in RPE cells, treating cells with oxidized photoreceptor outer segments will be considered in the following studies. Second, the protector effect at longer periods, as well as MADE effect after H_2_O_2_-induced oxidation will require further experiments. Third, an *in vivo* animal study and the efficacy of MADE in comparison with other drugs would need to be tested.

In summary, the present study proved that MADE is capable to protect RPE cells from H_2_O_2_-induced oxidative stress and apoptosis. The protective effect was mediated by inducing the activation of Nrf2/HO-1 signaling pathway. Considering the positive activity of MADE, we proposed that MADE might be explored as a therapeutic agent for the treatment of AMD.

## Abbreviations

AMD: age-related macular degeneration
bax: Bcl-2 associated X
bcl-2: B-cell lymphoma-2
CCK-8: cell counting kit-8
DCF: dichlorofluorescein
DCFH-DA: 2′,7′-dichlorofluorescein diacetate
DMEM/F12: Dulbecco's Modified Eagle Medium/F-12
GSH: glutathione
H_2_O_2_: hydrogen peroxide
MADE: madecassoside
MDA: malondialdehyde
Nrf2/HO-1: nuclear factor erythroid 2-related factor 2/heme oxygenase-1
ROS: reactive oxygen species
RPE: retinal pigment epithelium
siRNA: small interfering RNA
SOD: superoxide dismutase
