# Expression of Concern: Madecassoside protects retinal pigment epithelial cells against hydrogen peroxide induced oxidative stress and apoptosis through the activation of Nrf2/HO-1 pathway

**DOI:** 10.1042/BSR-2019-4347_EOC

**Published:** 2024-06-21

**Keywords:** Age‐related macular degeneration (AMD), madecassoside (MADE), oxidative stress, retinal pigment epithelium (RPE)

The Editorial Office has been made aware of potential issues surrounding the scientific validity of this paper, hence has issued an expression of concern to notify readers whilst the Editorial Office investigates. It has been noted that there appears to be similarities in the background details between some of the Western blots in this paper. Specifically, similarities between Figure 3B bcl-2 panel and Figure 3B β-actin panel, and separately similarities between Figure 4A nuclear Nrf2 panel and Figure 4A β-actin panel.

